# Identification and Characterization of Tunneling Nanotubes Involved in Human Mast Cell FcεRI-Mediated Apoptosis of Cancer Cells

**DOI:** 10.3390/cancers14122944

**Published:** 2022-06-14

**Authors:** Elnaz Ahani, Mohammad Fereydouni, Mona Motaghed, Christopher L. Kepley

**Affiliations:** 1 Department of Nanoengineering, Joint School of Nanoscience and Nanoengineering, North Carolina Agricultural and Technical State University, Greensboro, NC 27401, USA; eahani@aggies.ncat.edu (E.A.); mmotaghed@aggies.ncat.edu (M.M.); 2 Department of Nanoscience, Joint School of Nanoscience and Nanoengineering, University of North Carolina at Greensboro, Greensboro, NC 27401, USA; m_fereyd@uncg.edu; 3 Department of Molecular and Cellular Sciences, Liberty University College of Osteopathic Medicine, Lynchburg, VA 24502, USA

**Keywords:** mast cells, adipose-derived mast cells, IgE, tunneling nanotubes, cancer cells

## Abstract

**Simple Summary:**

Mast cells (MCs) are ubiquitously found in most tissues and in and around tumors. Their role in cancer pathogenesis remains an open area of investigation, and their interactions with tumor cells has not been explored. Here, a novel mechanism of communication between human MCs and tumor cells involving tunneling nanotubes (TnT) and other membrane structures is described. The formation of these communication structures is dependent on MC receptors interacting with tumor antigens through tumor-specific immunoglobulins and results in tumor-killing mediators from MC entering the tumor cells. This mechanism underlying the MC killing of tumor cells has important implications in understanding cancer pathogenesis.

**Abstract:**

Mast cells (MCs) are found in practically all tissues where they participate in innate and adaptive immune responses. They are also found in and around tumors, yet their interactions with cancer cells and the resulting impact on cancer cell growth and metastasis are not well understood. In this study, we examined a novel mechanism of IgE-FcεRI-mediated, intercellular communication between human adipose-derived mast cells (ADMC) and cancer cells. The formation of heterotypic tunneling nanotubes (TnT) and membrane structures between MCs and tumor cells in vitro was examined using microscopy and a diverse array of molecule-specific indicator dyes. We show that several MC-specific structures are dependent on the specific interactions between human tumor IgE-sensitized MCs and antigens on the tumor cell surface. The formation of TnT, membrane blebs and other MC-specific structures paralleled FcεRI-degranulation occurring within 30 min and persisting for up to 24 h. The TnT-specific adhesion of FcεRI-activated MCs to tumor cells was characterized by the transport of the MC granule content into the tumor cells, including tryptase and TNF-α. This interaction led to apoptosis of the tumor cells, which differs from previous studies examining tissue cells within the cancer microenvironment. The formation of heterotypic TnT results in stimulation of an invasive tumor cell phenotype and increased tumor cell invasion and chemoresistance of the cancer cells. These studies describe a heretofore-unrecognized mechanism underlying IgE-mediated interactions and FcεRI-activated MC-mediated killing of tumor cells through the formation of TnT.

## 1. Introduction

Mast cells (MCs) are most recognized as regulators of Type-I hypersensitivity following the release of allergic mediators through antigen binding to MCs bound IgE resulting in FcεRI-mediated activation and release of histamine. Mast cells are found in and around multiple tumor types, and numerous studies have assessed their numbers to correlate their presence with patient outcomes [1]. Yet, it is still unclear if they can induce pro- or figureanti-tumor functions, which has led to controversy regarding their role in cancer pathogenesis [2,3,4]. 

Nonetheless, human MCs have anti-tumor mediators that can be controlled to be released through FcεRI and induce apoptosis of cancer cells [5,6]. We found that tumor-targeted human MCs from adipose and peripheral blood bound to cancer cells and induced apoptosis in vitro through the FcεRI-mediated release of TNF-α [6]. In solid tumor human breast cancer (BC) xenograft mouse models, the infusion of HER2/*neu* IgE-sensitized human MCs co-localized to BC cells, decreased the tumor burden and prolonged the overall survival without indications of toxicity [7]. 

Specifically, tumor IgE-sensitized MCs were activated via FcεRI in a cell-number-dependent manner to release pre-stored and newly generated mediators that induced apoptosis of tumor cells. This cytotoxic effect of MCs was paralleled by the formation of several cell membrane protrusions, including what appeared to be TnT formed between MCs and tumor cells. Here, we further examine and define the kinetics of the formation of TnT between MCs and cancer cells.

TnT are cell-to-cell communication structures formed by filipodia-like membrane extensions that form connections between cells [8]. These projections are of great interest due to their ability to transport a wide range of molecules between cells [9,10]. An area of emerging research interest is TnT-mediated intercellular communication between cancer cells and those in tumor microenvironments. The TnT were initially observed between patient-derived cancer cell lines and resected solid tumors from patients and contributed to tumor heterogeneity, acquisition of an invasive phenotype, reprograming healthy neighboring cells and transferring mitochondria [9,10,11]. The transfer of mitochondria allows tumor cells to develop several parameters related to developing a cancer drug-resistant phenotype [10].

In this study, the structures that form from the interactions in co-cultures between FcεRI-activated human MCs and cancer cells were investigated. Several protrusions and formations emerged between the cells, which were mediated by a tumor recognizing IgE binding to the requisite surface antigens on tumor cells. These structures include TnT, membrane blebs and other MC-specific protrusions. 

The binding of MCs to tumor cells resulted in the MC penetrating into the cancer cells where degranulation was followed by the formation of cancer cell apoptotic bodies. MC-specific mediators were shown to be released into the cancer cells following IgE–Ag binding, which was paralleled by the formation of MC membrane structures. These studies revealed a heretofore-unrecognized anti-tumor mechanism of direct intercellular exchange as a modulator of tumor apoptosis by IgE-sensitized human MCs.

## 2. Materials and Methods

### 2.1. Scanning Electron Microscopy

Adherent HER2/*neu*-positive cancer cell lines BT-474 and SK-BR-3 cells (ATCC, Manassas, VA) were removed from the flask surface using Cellstripper^TM^, pelleted, filtered (40 μm) and re-seeded on EM coverslip bottoms in six-well plates to attach and grow for ~72 h. Human ADMC (1.5 × 10^5^) [6] were collected and sensitized with or without 1 μg/mL of anti-HER2/*neu* (Absolute Antibody, Boston, MA) IgE or non-specific IgE (psIgE) for 2 h. After washing, filtered ADMC were added to HER2/*neu*-positive BT-474 (50–60% confluent) and incubated for the indicated times at 37 °C, washed with PBS and fixed with 2.5% glutaraldehyde and 4% formaldehyde in PBS for 2 h. 

Following three rinses with distilled water, the samples were dehydrated through a gradient series of ethanol (50%, 70%, 80%, 90% and 100%). After supercritical drying specimens were mounted on stubs using conductive double-sided carbon tape and coated with 12 nm thick gold-palladium by a sputter coater (Leica Microsystems, IL, USA). Cells were examined using a field emission scanning electron microscope (Zeiss Auriga FIBFESEM, Zeiss, New York, NY, USA) at 4 kV. All experiments were performed in duplicate from three to four separate donors, and apoptosis was measured as described [6].

### 2.2. Atomic Force Microscopy (AFM) of TnT

Atomic force microscopy was used to assess the formation of TnT between MCs and BT-474 cells. Anti-HER2/*neu* IgE-sensitized MCs were prepared as above. After 24 h of incubation, co-cultured cells were washed with PBS, fixed with 2% glutaraldehyde in PBS to cover the surface for 5 min and post-fixed with paraformaldehyde (4% in PBS) for 30 min at room temperature. After washing, cells were examined using AFM (Asylum MFP-3D Origin+ AFM, Santa Barbara, CA, USA).

### 2.3. Live-Cell Imaging of Heterotypic TnT between MCs and Cancer Cells Using Confocal Microscopy

The formation of TnT between ADMC and cancer cells was monitored by co-culturing the HER2/*neu*- positive BT-474 or SK-BR-3 cells using confocal microscopy with differentially labeled cells. HER2/*neu* or psIgE-sensitized MCs (1 × 10^5^) were cultured in X-VIVO with SCF (80 ng/mL) overnight in a live cell incubator attached to a confocal microscope. CellTracker™ Deep Red (1 μM; InvitroGen, Waltham, MA) and CellBrite^®^ NIR cytoplasmic membrane dye (Biotium, Fremont, CA; 2 μM) were added to ADMC for 1 h, washed and processed as below in different experiments. In some experiments, co-cultures were incubated at the indicated times, and live-cell imaging was performed. 

For fixed cell imaging, 4% paraformaldehyde in PBS was added to the cells at 37 °C for 45 min, washed and wheat germ agglutinin (WGA, 1 μM, Molecular Probes, Invitrogen) was added to co-cultured cells. Cells were permeabilized using 0.2% Triton X-100 in PBS for 15 min and washed with PBS. In some experiments, cells were stained with Alexa Fluor 488^®^ phalloidin or Alexa Fluor 647^®^ phalloidin (1 μM, Molecular Probes, Invitrogen) at room temperature for 1 h. 

Cells were washed three times with PBS to remove excess dye, coverslips mounted in SlowFade Diamond Antifade mount (Molecular Probes, Invitrogen) and cells imaged on a microscope using the appropriate filter set for each dye combination. Images were acquired at 10-min intervals for up to 5 days at 100× magnification. Analysis of captured images was performed ImageJ software (National Institutes of Health, http://imagej.nih.gov/ij/, accessed on 19 April 2022). All experiments were performed in duplicate from three to four separate donors, and apoptosis was measured as described [6].

### 2.4. TnT Quantitation

The formation of TnT was assessed following the addition of psIgE or HER2/*neu*-sensitized MCs to BT-474 cells as above for 24 h. The TnT formations were quantified from randomly picked cells (*n* = 25) for each condition and are presented as dot plots with individual values of the number of TnT connections for each of three independent experiments as well as the overall mean of all experiments.

#### Transfer of MC Granule Contents into Cancer Cells

BT-474 or SK-BR-3 cells were grown on confocal slides (50–60% confluent), labeled with MitoTracker™ Green FM (1 μM) or CellTracker™ Deep Red (1 μM) for 1 h and washed three times in PBS. The labeled or unlabeled cancer cells were co-cultured with Hoechst-stained and unstained HER2/*neu* or psIgE-sensitized MCs (1 × 10^5^) for the indicated times in different experiments. Co-cultures were washed, fixed in cold methanol, blocked with 1:100 normal goat serum and permeabilized with 0.2% Triton X-100 in PBS for 15 min. Then, they were incubated with mouse anti-human tryptase, TNF-α or control; non-specific mouse IgG Abs (MOPC; 5 μg/mL) at 4 °C overnight. 

Slides were rinsed with PBS, incubated with secondary Abs using donkey anti-mouse IgG (Cy5^®^, 1 μM, Thermo Fisher, Waltham, MA, USA) for 1 h in a dark chamber at room temperature. Slides were washed and imaged for the indicated times by bright-field or fluorescent confocal microscopy using the appropriate fluorescent filter sets. The staining of the tryptase and TNF-α Abs was determined to be specific as non-specific mouse IgG Abs (MOPC) were not immune-reactive with the dye-labeled secondary Abs (see Figures S1–S4). All experiments were performed in duplicate from three to four separate donors.

## 3. Results

### 3.1. Mast Cell Binding to Cancer Cells Involves FcεRI-Dependent Formation of TnT

**Figure 1:**
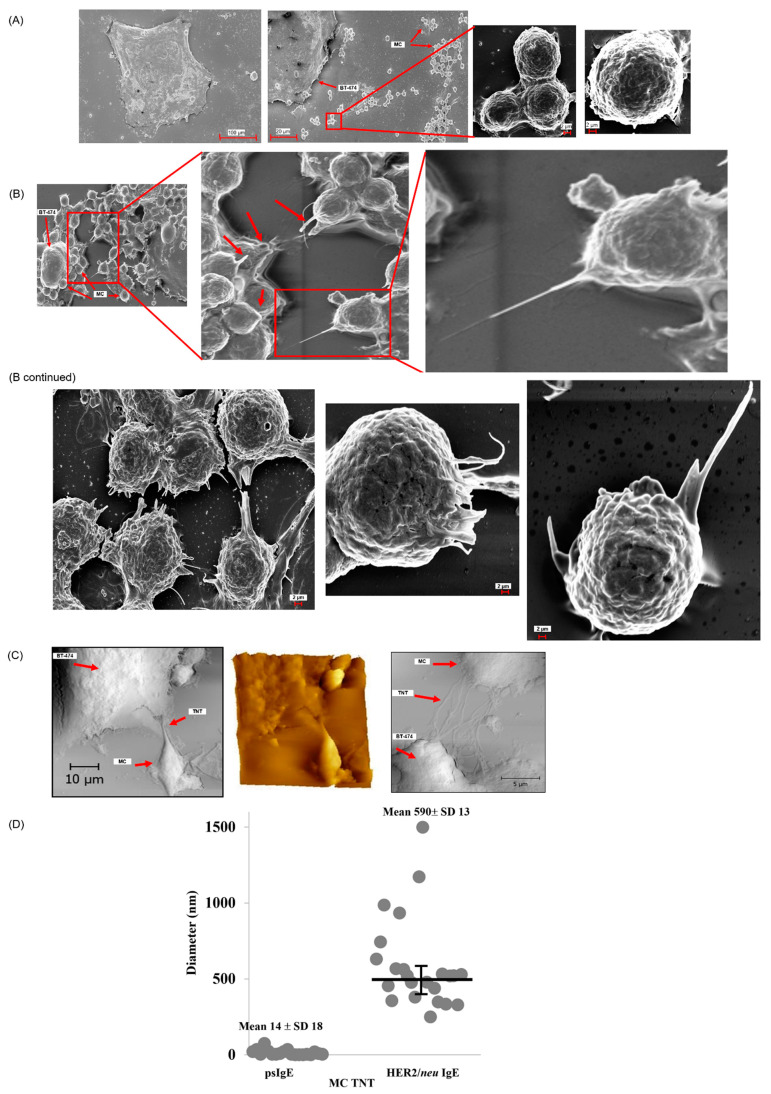
MC binding to HER2/*neu*-positive cancer cells is IgE dependent. *Note.* ADMC (1.5 × 10^5^) were sensitized without (**A**) or with (**B**) human anti-HER2/*neu* IgE, washed and incubated with BT-474 cells for 72 h. Cells were then processed for SEM imaging. (**C**) For further characterization of TnT formation, HER2/*neu* IgE-sensitized MCs were co-cultured with BT-474 cells, and the preparation steps were done for AFM imaging. (**D**) Mean ± s.e.m. distribution of TnT diameters from images between HER2/*neu* IgE-sensitized ADMC and BT-474 cells. *n* = 25 TnT.

We showed previously that HER2/neu IgE-sensitized human MCs can induce apoptosis of HER2/neu expressing BC cells [6]. However, the nature of the physical association between these cell types in mediating this response has not been explored. Thus, we sought to further examine this interaction between MCs and BC cells using electron microscopy. As seen in Figure 1A, MCs sensitized with psIgE demonstrated very little binding to the BT-474 cells with few ruffles or projections emerging from their membranes. Many psIgE MCs are seen far from BT-474 cells suggesting TnT mediate the FcεRI-mediated binding. 

The HER2/neu IgE-sensitized MC binding to HER2/neu-positive BT-474 cells were characterized by protrusions, characteristic of TnT, some of which attach to the BC cells (Figure 1B). AFM was also used to assess the formation of TnT. Figure 1C clearly shows that TnT form between HER2/neu IgE-sensitized MCs and BT-474 cells. Quantitation of the psIgE-sensitized and HER2/neu-sensitized MCs demonstrates that heterotypic TnT emerging from the MC following IgE crosslinking have an average diameter of ∼590 nm, with a broad range in individual diameters compared to non-FcεRI activated MC (Figure 1D). These results further indicate HER2/neu IgE regulates MC binding and activation when co-cultured with BT-474 cells. This process is partly controlled by the formation of MC TnT that attach to the BT-474 cells following HER2/neu IgE-FcεRI receptor binding.

### 3.2. Kinetics of MC FcεRI-Mediated TnT Formation

**Figure 2:**
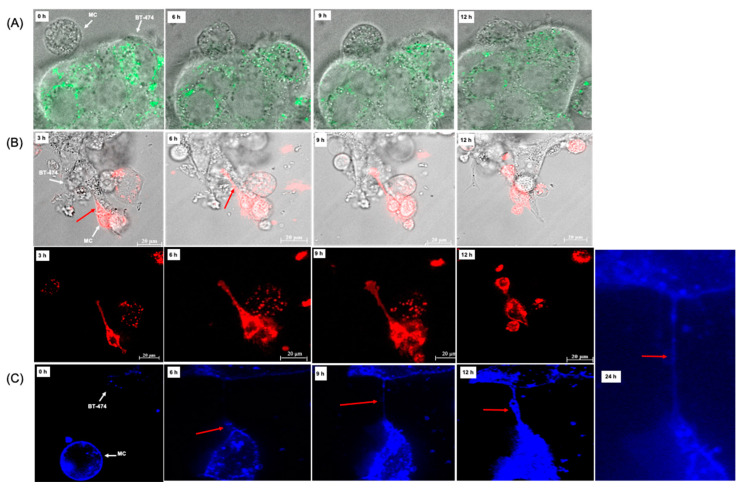

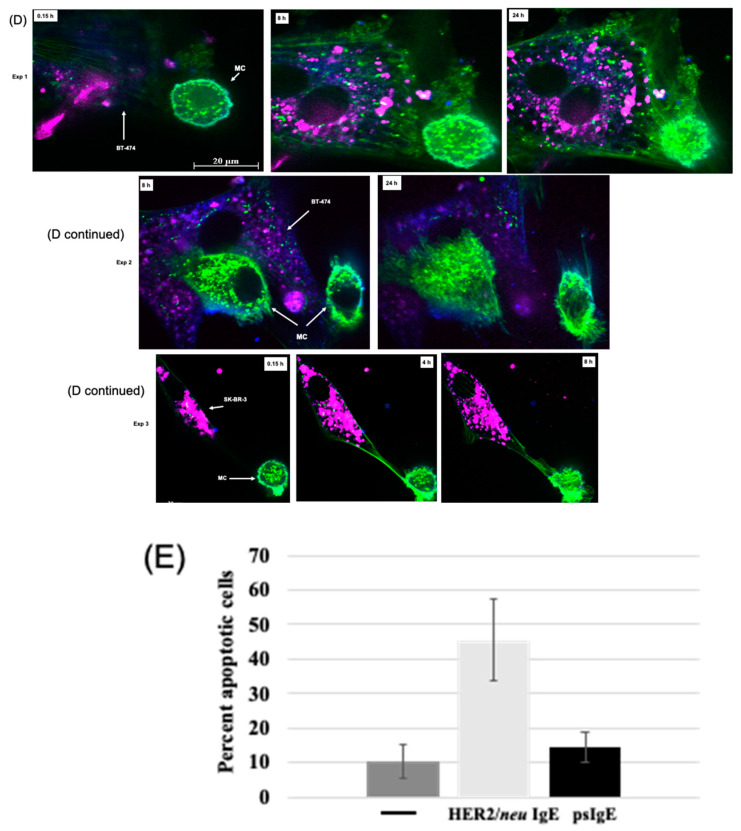
MC binding, penetration and apoptosis of BT-474 cells involve TnT. *Note.* (**A**) BT-474 cells were grown on confocal slides (50–60% confluent) and MitoTracker™ Green FM was added to the cells. In (**B**) ADMC (1 × 10^5^) sensitized with HER2/*neu* IgE were labeled with CellBrite^®^, washed and added to the unlabeled BT-474 cells and visualized for the indicated times by bright-field and fluorescent microscopy. In (**C**,**D**), co-cultures were incubated for the indicated times, washed, fixed and WGA added as described in the Materials and Methods section. Cells were visualized with fluorescent microscopy. (**E**) Quantification of overall apoptosis comparing psIgE and anti-HER2/*neu* IgE-sensitized cells at Day 4 from three experiments. Mean ± sd.

We used co-culture experiments to more extensively investigate the binding and interaction of the MCs and cancer cells more extensively using a variety of dye-labeling strategies. As seen in Figure 2A unstained HER2/*neu* IgE-sensitized MCs bound to and penetrated MitoTracker™ Green-FM-labeled HER2/*neu*^+^ BT-474. Only the IgE-FcεRI-activated MC produced TnT as psIgE MC did not bind to the tumor cells or form TnT (Figures S1 and S2 and Video S1). The TnT appeared within 1 h and persisted up to four days. HER2/*neu* IgE-sensitized MCs labeled with CellBrite^®^ (Figure 2B) or WGA (Figure 2C) demonstrated the formation of TnT from the plasma membrane of MCs following FcεRI-bound IgE binding to BC antigens. 

In some experiments, the MCs made contact, penetrated and appeared to form a new MCs within the cancer cell, as shown by bright-field (top) and fluorescent (bottom) microscopy (Figure 2B and Video S2). The FcεRI dependent TnT reacted with WGA, suggesting that they contain plasma membrane N-acetyl-D-glucosamine and/or sialic-acid-containing glycoconjugates and oligosaccharides (Figure 2C). Differential labeling of the BC cells (CellBrite^®^) and MCs (MitoTracker™ Green FM) revealed the TnT formed from the MCs (Figure 2D), which paralleled BC cell apoptosis (Figure 2E). 

The FcεRI-activated MCs had multiple cancer cell engagement points that mediated the attachment and spreading of the MCs. The MCs had significant changes in morphology as they spread across and into the cancer cells. Time-lapse video of MitoTracker™ Green-FM-labeled MCs confirmed the formation and attachment of TnT to the BC cells as seen in Video S3 (BT-474) and Video S4 (SK-BR-3). These experiments further suggest that MCs induce the FcεRI-dependent apoptosis of cancer cells through direct cell binding by MC TnT, and they provide new insights into the physiological manifestations of MC killing of tumor cells.

### 3.3. Human MCs form Membrane Blebs upon FcεRI Activation by Cancer Cells

**Figure 3:**
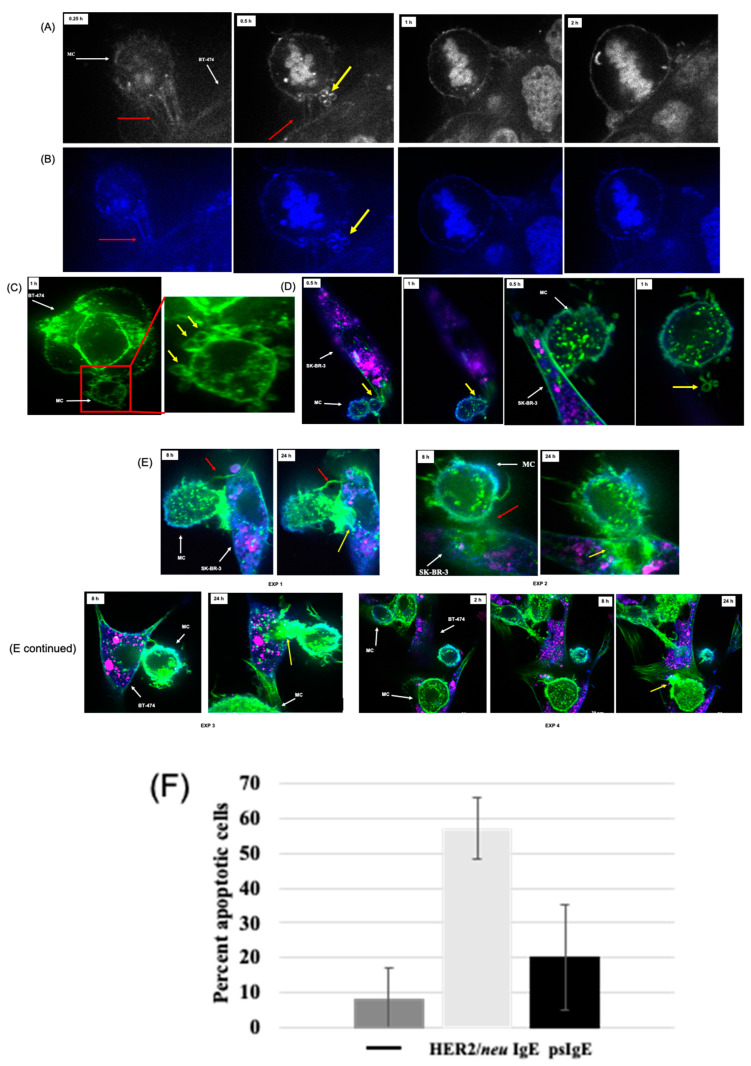
IgE-dependent formation of TnT and membrane blebs between MCs and BC cells. Note. BT-474 or SK-BR3 cells were grown on confocal slides (50–60% confluent). ADMC (1 × 10^5^) with HER2/*neu* IgE were labeled with WGA (**A**,**B**) or MitoTracker™ Green-FM-labeled (**C**–**E**), washed and added to the cancer cells (described in the Materials and Methods section) and visualized with black and white and color fluorescent microscopy. (**F**) Quantification of overall apoptosis comparing psIgE and anti-HER2/*neu* IgE-sensitized cells at Day 4 from three experiments. Mean ± sd.

We further investigated the interaction between MCs and BT-474 cells up to 24 h following FcεRI activation. The HER2/*neu* IgE-sensitized MCs began forming TnT that attached to the HER2/*neu*-positive BT-474 cells within 15 min (Figure 3A,B; red arrows). There were also vesicular, membrane blebbing substructures that stained with WGA at 0.5 h and visualized by black and white fluorescent microscopy (Figure 3A; second column, yellow arrows and Video S5). 

Blebbing substructures that stained with WGA and Alexa Fluor 488^®^ phalloidin were imaged by fluorescent microscopy, respectively (Figure 3B,C). The membrane blebs also appeared between MitoTracker™ Green-FM-labeled, HER2/*neu* IgE-sensitized MCs and CellBrite^®^ stained SK-BR-3 cells at various times and only during FcεRI activation (Figure 3D,E), which paralleled BC cell apoptosis (Figure 3F).

### 3.4. IgE-Dependent Transfer of Anti-Tumor Mediators from MCs into Cancer Cells

The formation of TnT that transfer cellular material between cancer cells and immune cells has been described previously using other cell types [8]. To assess if FcεRI- activated MCs transferred mediators into the cancer cells, experiments were performed using co-cultures followed by IHC detection of MC-specific mediators. 

**Figure 4:**
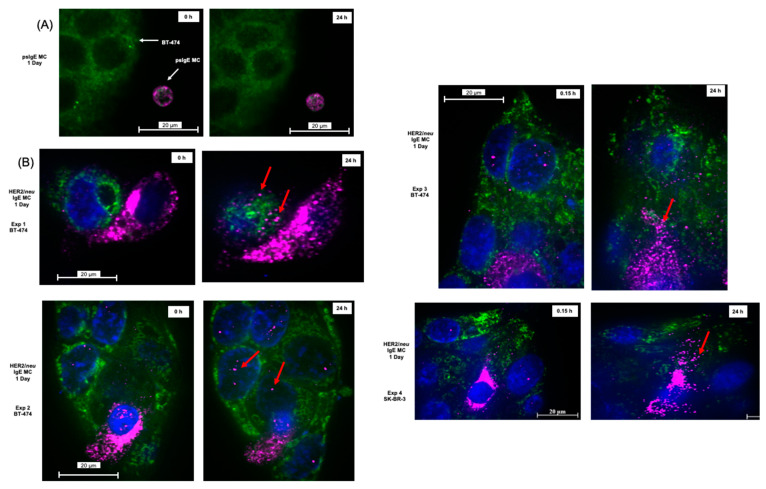

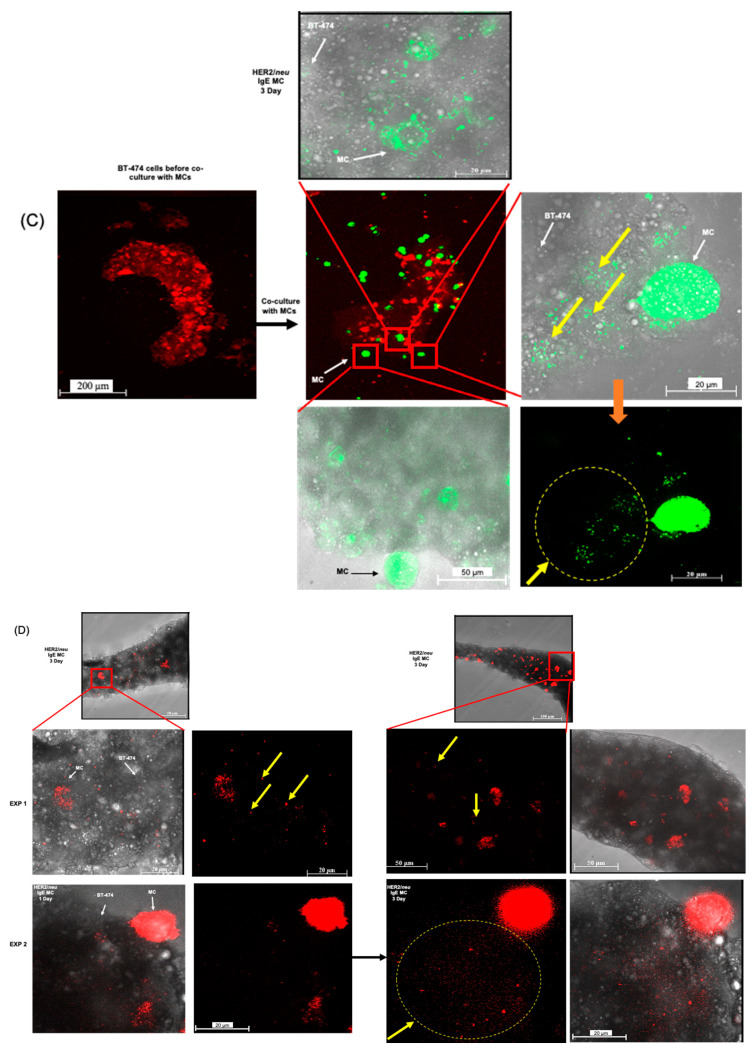
The release of granule mediators MCs into cancer cells is FcεRI dependent. Note. Labeled BT-474 or SK-BR-3 cells were grown on confocal slides (50–60% confluent), and psIgE or HER2/*neu*-sensitized ADMC (1 × 10^5^) were added for the indicated times (**A**–**C**). Slides were fixed and labeled with anti-tryptase (**A**–**C**) and followed by secondary Ab as described in Materials and Methods section. (**D**) HER2/*neu*-sensitized ADMC (1 × 10^5^) were added to unlabeled BT-474 cells for the indicated times, fixed and labeled with anti-TNF-α Ab (1 μg/mL) followed by secondary Ab. Experiments are representative of experiments performed from three to four different donors.

Using psIgE-sensitized MCs, there was no formation of TnT, attachment of MC to BT-474, or transfer of material (Figure 4A). In contrast, MCs sensitized with HER2/*neu* clearly degranulated and transferred the MC-specific protease tryptase into the cancer cells over 24 h and 72 h as detected using IHC (Figure 4B,C). The specificity of the IHC staining using a non-specific control (MOPC) for the anti-tryptase demonstrated no immunoreactivity (Figure S3). TNF-α is another anti-tumor mediator uniquely stored in MC granules [12] and critical for apoptosis of tumor cells following FcεRI activation [6]. We investigated if tumor-triggered MCs could transfer TNF-α into tumor cells following the formation of TnT. 

As seen in Figure 4D, HER2/*neu* IgE-sensitized MCs were activated by BT-474 cells to release TNF-α into the tumor cells at three days as detected with TNF-α -specific Abs. The TNF-α was scattered randomly and diffusely within the tumor cells and only in those co-cultured with MCs sensitized with HER2/*neu* Abs. As predicted, no TNF-α was detected in cancer cells co-cultured with psIgE-sensitized MCs (Figure S4). These data suggest tumor IgE-targeted MCs can bind to requisite tumor cells with markers targeted by the IgE to release pre-formed granule mediators into the tumor cells.

## 4. Discussion

Adipose-derived, tumor-specific IgE-sensitized human MCs were previously shown to bind to and induce apoptosis of tumor cells in vitro [6] and in vivo [7]. The studies showed that IgE-FcεRI-activated MCs form several structures in vitro that appear to mediate the transfer of granule contents into cancer cells through an FcεRI-mediated mechanism. 

The formation of these structures includes TnT-like protrusions containing N-acetylglucosamine and/or sialic acid and membrane blebs that appear within 1 h following FcεRI activation and coincide with the release of MC granule contents into the cancer cells. This interaction led to apoptosis of the tumor cells, which differs from previous studies examining tissue cells within the cancer microenvironment. The formation of heterotypic TnT results in stimulation of an invasive tumor cell phenotype, increased tumor cell invasion and chemoresistance of the cancer cells [13,14]. This may be part of the FcεRI apoptosis-inducing mechanism MCs use to kill cancer cells described previously [6].

The formation of TnT has been demonstrated for a wide range of cells and allowed for cell–cell communication. These are the first reports of human FcεRI-activated MCs forming TnT that mediate granule content exchange into cancer cells. These MC-derived structures are only formed after IgE-FcεRI activation and were characterized according to guidelines outlined recently, including the morphological and 3D structure identification using SEM and cytoskeleton component identification and associated elements (e.g., WGA) using confocal microscopy [15]. 

Specifically, our approach allowed us to monitor and quantify the MC TnT formation through electron, atomic force, bright-field and fluorescent confocal microscopy using an array of organelle and molecule-specific dyes. Second, we were able to quantify numbers, lengths and z position of TnT from MCs using confocal microscopy and organelle/molecule-specific dyes. Third, the average size of the TnT from MCs was similar as reported in other studies using other tissue cells [14]. Fourth, the MC TnT did not appear to make contact with the substrate of the culture dish, another characteristic defined previously [15].

Previous studies using a MC line, LAD2, reported the emergence of vesicle-like structures when co-cultured with glioblastoma cells [16]. These transformed immortal cells were not challenged through FcεRI as performed with primary human MCs described here. Currently, we are investigating if the MCs form TnT in vivo using patient-derived xenograft models in immunocompromised mice to determine any correlations between TnT formation and their ability to induce tumor shrinkage.

These studies describe for the first-time membrane blebs that form on FcεRI-activated primary human MCs, which parallels apoptosis of tumor cells. Membrane blebbing has been described before on several cell types and occurs when the cell membranes break from cellular anchor proteins causing the membrane protrusions to form due to the release of intracellular pressure during cytokinesis and cell migration [17]. 

Previous studies using a transformed rodent cell line that can be activated through FcεRI showed membrane blebs forming in medium contain IgE-dependent and IgE-independent stimuli [18,19]. The large, non-detaching, non-apoptotic, membrane structures were similar to those we observed (Figure 3). We observed the membrane blebbing between two separate tumor cells and preceded or closely paralleled tumor cell binding of the MCs through TnT and tumor cell apoptosis. The biological significance of MC membrane blebs has not been elucidated, and it is not known if they form in vivo.

Our lab is pursuing the use of autologous human MCs as a new strategy for cancer immunotherapy [6,20]. One anti-tumor mediator uniquely prestored and released from MCs through FcεRI stimulation is TNF-α [6,12,21]. TNF-α has been extensively investigated as an anti-cancer agent and adjuvant [22,23,24,25,26,27,28]. The biggest impediment to utilizing the anti-tumor properties of TNF-α is its systemic, non-target effects. Finding ways to focus and maximize the local concentration targeting tumor cells and minimizing the dose may hold promise in anti-tumor therapies in which tumor invasion by adoptive cell transfer limits the solid tumor efficacy [29]. 

Here, evidence is presented that tumor-targeted MCs may be one solution to this problem. First, MCs form TnT with the cancer cells and appear to transfer TNF-α specifically to the tumor cell. Second, we observed that MCs are completely opsonized within tumor cells where they continue their release of granule content (Figure 2, Figure 3 and Figure 4). 

Subsequently, the characteristic markers of apoptosis are evident following the involution of the MC within BT-474 and SK-BR-3 cells resulting in cytoplasm swelling and formation of apoptotic bodies, as we demonstrated previously with these two cell lines [6]. This could be one especially beneficial aspect for the use of MCs as an autologous source for cell-based cancer immunotherapy by ensuring the TNF-α only enters the cancer cells attached to the MCs and is not released systemically.

As with TNF-α, tryptase is uniquely pre-stored in MC granules [30], released upon IgE-FcεRI crosslinking and enters the cancer cells following their IgE-mediated interaction with MCs. The mechanistic role of tryptase in tumor growth and metastasis is unknown. Many of the correlative studies associating a pro- or anti-tumor role of MCs based on an increase or decrease in their numbers have used tryptase as the MC-specific marker [3]. It has been suspected, but not definitively established, that MC-derived tryptase (and chymase) may induce tumor angiogenesis. However, the majority of these studies use in vitro assays that combine tryptase inhibitors with tryptase-challenged endothelial cells as a model for vascular growth [30,31]. 

In general, links to angiogenesis are attributed to the ability of tryptase to degrade or activate substrates involved in angiogenesis (e.g., fibrinogen and collagenase) [32]. Other studies suggest the opposite role of MCs in tumor growth. MC-derived tryptase binds to exosomes released from tumor cells and is taken up into the nucleus where it inhibits cell proliferation [33]. While these studies demonstrate these MC mediators are transferred into cancer cells, it is still unclear which one mediates apoptosis. Studies using MCs that have been selectively depleted of certain mediators are underway to determine which mediators are pro- or anti-apoptotic. 

## 5. Conclusions

In conclusion, these studies describe a new cross-talk mechanism between MCs and cancer cells controlled by the FcεRI pathway. The formation of TnT between MC and cancer cells may have important implications in the MC-induced apoptosis observed. This also demonstrates an advantage of this strategy of using autologous MCs as anti-tumor cells since anti-tumor mediators, such as TNF-α, can be targeted and released through a tightly controlled mechanism such that the molecules are delivered specifically within the tumor cells. TnT may also mediate FcεRI-mediated communication in the context of cell-to-cell interactions and suggests that further studies are warranted to assess TnT formation in vivo, understand the mechanisms underlying their formation and identify other molecules/organelles transferred between tumor IgE-sensitized MCs and tumor cells.

## Data Availability

This article includes all data generated or analyzed during this study.

## Supplementary Materials

The following supporting information can be downloaded at: https://www.mdpi.com/article/10.3390/cancers14122944/s1, Figure S1: Fluorescent microscopy of psIgE MCs with BT-474 cells in a co-culture system (Control). MitoTracker™ Green-FM-labeled BT-474 (S1) were added to CellBrite^®^-labeled ADMC (1 × 105) sensitized with psIgE for the indicated times. Figure S2: Fluorescent microscopy of psIgE MCs with SK-BR-3 cells in a co-culture system (Control). CellBrite^®^-labeled SK-BR-3 were added to MitoTracker™ Green-FM-labeled ADMC (1 × 105) sensitized with psIgE for the indicated times. Figure S3: Immunohistochemistry of non-specific control (MOPC) for tryptase experiments (Control). Hoechst-stained HER2/*neu*-sensitized ADMC (1 × 105) were added to the MitoTracker™ Green FM-labeled BT-474 and SK-BR-3 cells for three days. Slides were fixed and labeled with MOPC followed by secondary Abs. Figure S4: Immunohistochemistry of psIgE and non-specific control (MOPC) for anti-TNF-α experiments (Control). (Left) psIgE ADMC were added to the BT-474 cells for three days. Slides were fixed and labeled with anti-TNF-α Ab followed by secondary Ab. (Right) HER2/*neu* ADMC were added to the BT-474 cells for three days. Slides were fixed and labeled with MOPC followed by secondary Ab. Video S1. TnT are not formed between psIgE-sensitized MCs and BT-474 cells. BT-474 cells were grown on confocal slides (50–60% confluent). The psIgE MCs were labeled with CellBrite^®^ and were added to BT-474 cells, and a time-lapse video was recorded for 24 h by fluorescent microscopy. The results showed no or minimal interaction between MCs and BC cells. Video S2. Fluorescent microscopy of the interaction of MCs with BC cells. HER2/*neu* IgE-sensitized MCs were labeled with CellBrite^®^ and added to BT474 cells. After co-culturing, MCs formed TnT on the cancer cells and infiltrated into the BT-474 cells. Time-lapse video was acquired for 24 h of co-culture using fluorescent microscopy. Video S3. MC binding to BT-474 cells involves TnT. BT-474 cells were grown on confocal slides (50–60% confluent) and were labeled with CellBrite^®^. MCs (1 × 105) were labeled with MitoTracker™ Green and sensitized with HER2/*neu* IgE. Then, after fixation, they were labeled with WGA- Alexa Fluor 488^®^ phalloidin (as described in Materials and Methods section), and time-lapse videos were acquired for 24 h of co-culture by fluorescent microscopy. Video S4. MC binding to SK-BR-3 cells involves TnT. SK-BR-3 cells were labeled with CellBrite^®^ and were added to HER2/*neu* IgE MCs (1 × 105) labeled with MitoTracker™ Green. Then, after fixation, they were labeled with WGA- Alexa Fluor 488^®^ phalloidin (as described in Materials and Methods section), and time-lapse videos were acquired for 24 h of co-culture by fluorescent microscopy. Video S5. Formation of TnT and membrane blebs between MCs and BT-474 cells. MCs were sensitized with HER2/*neu* IgE and then added to BT-474 cells. Time-lapse video was acquired for 24 h of co-culture by fluorescent microscopy (in a black and white view) to show MC TnT and membrane blebs forming with cancer cells.

## Abbreviations

Abbreviation	Meaning
MCs	Mast cells
BC	Breast cancer
ADMC	Adipose-derived mast cells
HER2/*neu*	Human epidermal growth factor receptor 2
FcεRI	High-affinity receptor for IgE
IgE	Immunoglobin E
TNF-α	Tumor necrosis factor alpha
GM-CSF	Granulocyte-macrophage colony-stimulating factor
TnT	Tunneling nanotubes
WGA	Wheat Germ Agglutinin
Ab	Antibody
Ag	Antigen
