# Supplementary material for: Identification and Characterization of Tunneling Nanotubes Involved in Human Mast Cell FcεRI-Mediated Apoptosis of Cancer Cells
Source: Cancers (Basel). 2022 Jun 14;14(12):2944. doi: 10.3390/cancers14122944 (PMC9220880; doi:10.3390/cancers14122944)
Supplement: Supplementary file 1 [file cancers-14-02944-s001.zip › cancers-1714124-supplementary.pdf]

cancers

Supplementary Materials

Identification and Characterization of Tunneling Nanotubes Involved in Human Mast Cell FcεRI-Mediated Apoptosis of Cancer Cells

Elnaz Ahani [1], Mohammad Fereydouni [2], Mona Motaghed [1] and Christopher L. Kepley [3,*]

[1] Department of Nanoengineering, Joint School of Nanoscience and Nanoengineering, North Carolina Agricultural and Technical State University, Greensboro, NC 27401, USA; eahani@aggies.ncat.edu (E.A.); mmotaghed@aggies.ncat.edu (M.M.)
[2] Department of Nanoscience, Joint School of Nanoscience and Nanoengineering, University of North Carolina at Greensboro, Greensboro, NC 27401, USA; m_fereyd@uncg.edu
[3] Department of Molecular and Cellular Sciences, Liberty University College of Osteopathic Medicine, Lynchburg, VA 24502, USA
* Correspondence: ckepley@liberty.edu; Tel.: +1-540-808-8993

(S1)

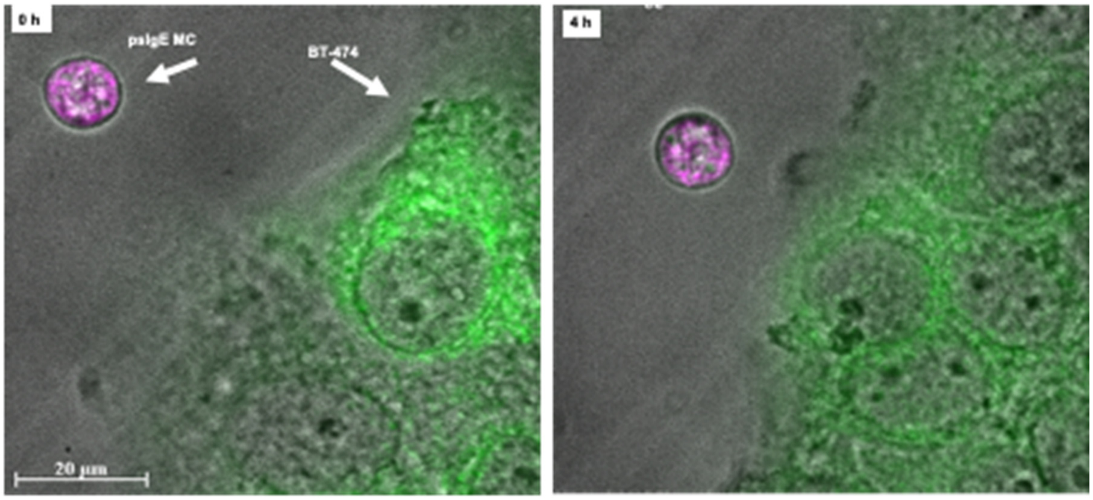

Figure S1. Fluorescent microscopy of psIgE MCs with BT-474 cells in a co-culture system (Control). MitoTracker™ Green-FM-labeled BT-474 (S1) were added to CellBrite®-labeled ADMC (1 × 10^5) sensitized with psIgE for the indicated times.

(S2)

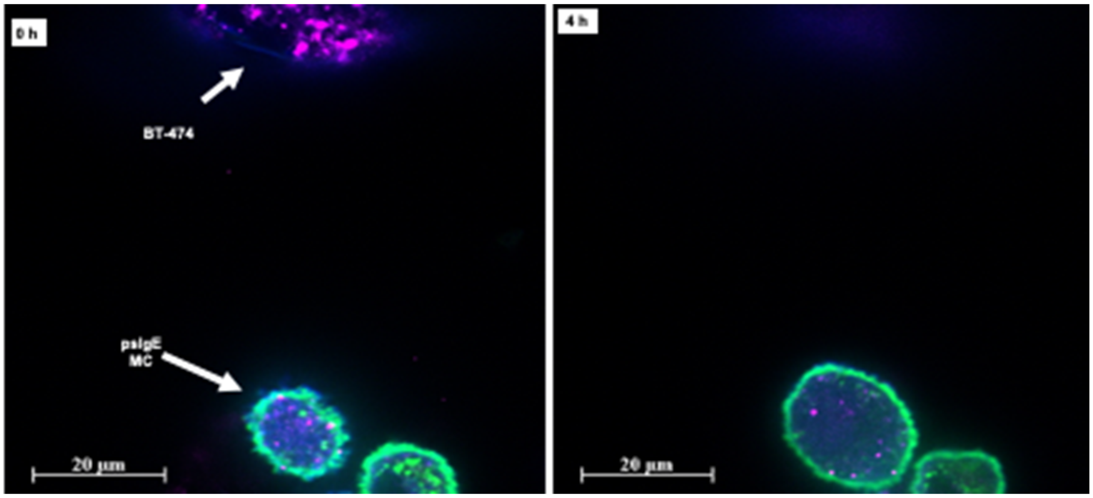

Figure S2. Fluorescent microscopy of psIgE MCs with SK-BR-3 cells in a co-culture system (Control). CellBrite®-labeled SK-BR-3 were added to MitoTracker™ Green-FM-labeled ADMC (1 × 105) sensitized with psIgE for the indicated times.

(S3)

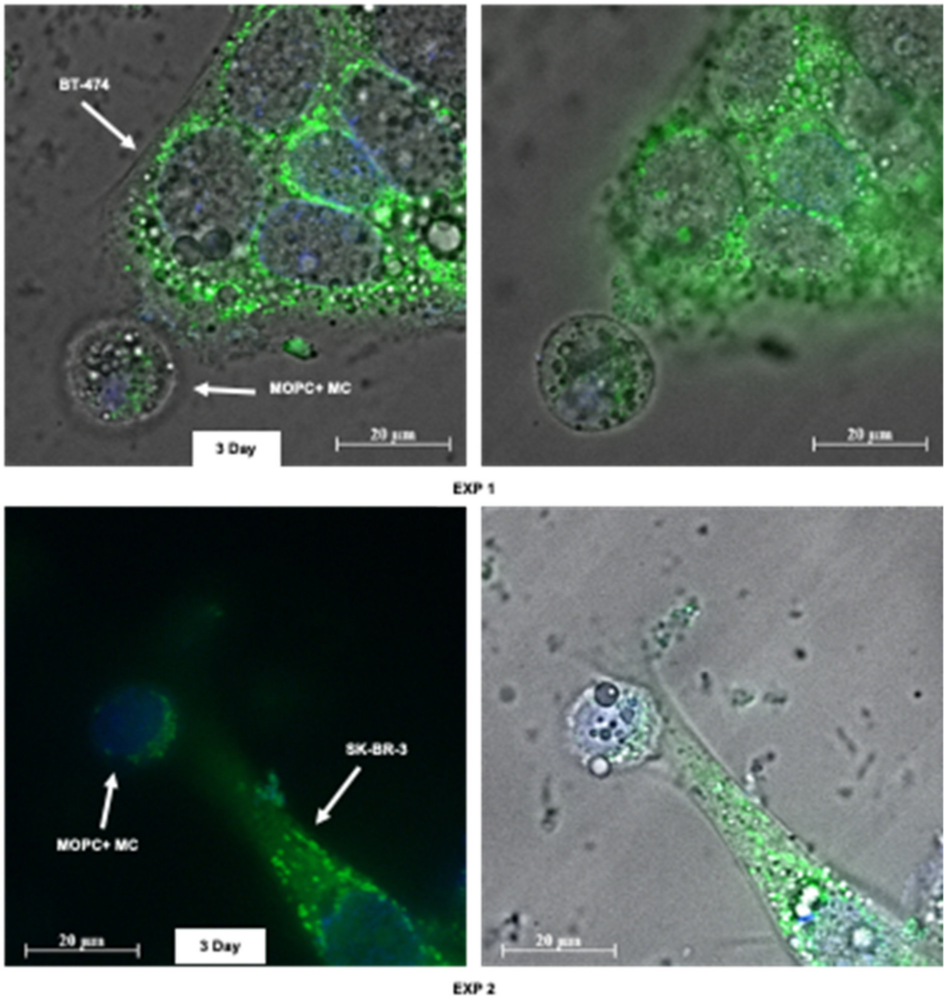

Figure S3. Immunohistochemistry of non-specific control (MOPC) for tryptase experiments (Control). Hoechst-stained HER2/neu-sensitized ADMC (1 × 105) were added to the MitoTracker™

Green FM-labeled BT-474 and SK-BR-3 cells for three days. Slides were fixed and labeled with MOPC followed by secondary Abs.

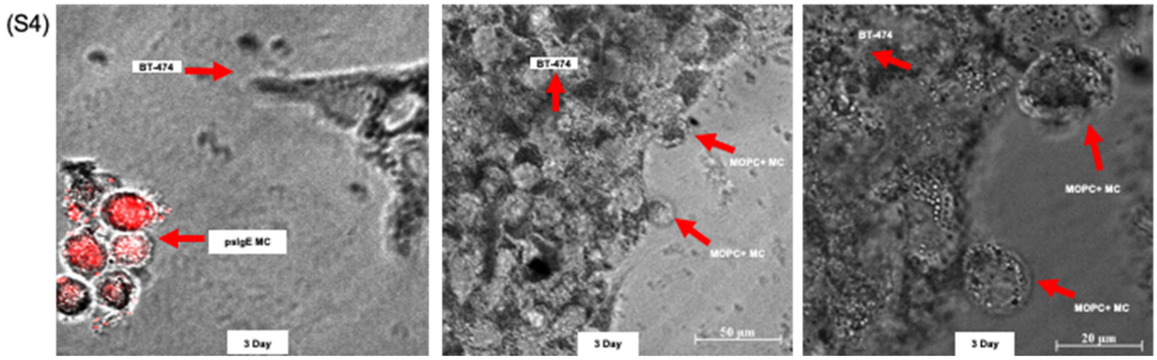

Figure S4. Immunohistochemistry of psIgE and non-specific control (MOPC) for anti-TNF-α experiments (Control). (Left) psIgE ADMC were added to the BT-474 cells for three days. Slides were fixed and labeled with anti-TNF-α Ab followed by secondary Ab. (Right) HER2/neu ADMC were added to the BT-474 cells for three days. Slides were fixed and labeled with MOPC fol-lowed by secondary Ab.

Legend for Videos:

Video (S1). TnT are not formed between psIgE-sensitized MCs and BT-474 cells. BT-474 cells were grown on confocal slides (50–60% confluent). The psIgE MCs were la-beled with CellBrite® and were added to BT-474 cells, and a time-lapse video was recorded for 24 h by fluorescent microscopy. The results showed no or minimal interaction between MCs and BC cells.

Video (S2). Fluorescent microscopy of the interaction of MCs with BC cells. HER2/neu IgE-sensitized MCs were labeled with CellBrite® and added to BT474 cells. After co-culturing, MCs formed TnT on the cancer cells and infiltrated into the BT-474 cells. Time-lapse video was acquired for 24 h of co-culture using fluorescent microscopy.

Video (S3). MC binding to BT-474 cells involves TnT. BT-474 cells were grown on confocal slides (50–60% confluent) and were labeled with CellBrite®. MCs (1 × 105) were labeled with MitoTracker™ Green and sensitized with HER2/neu IgE. Then, after fixation, they were labeled with WGA- Alexa Fluor 488® phalloidin (as described in Materials and Methods section), and time-lapse videos were acquired for 24 h of co-culture by fluorescent microscopy.

Video (S4). MC binding to SK-BR-3 cells involves TnT. SK-BR-3 cells were labeled with CellBrite® and were added to HER2/neu IgE MCs (1 × 105) labeled with Mito-Tracker™ Green. Then, after fixation, they were labeled with WGA- Alexa Fluor 488® phalloidin (as described in Materials and Methods section), and time-lapse videos were acquired for 24 h of co-culture by fluorescent microscopy.

Video (S5). Formation of TnT and membrane blebs between MCs and BT-474 cells. MCs were sensitized with HER2/neu IgE and then added to BT-474 cells. Time-lapse video was acquired for 24 h of co-culture by fluorescent microscopy (in a black and white view) to show MC TnT and membrane blebs forming with cancer cells.
